# Closed-Form UAV LoS Blockage Probability in Mixed Ground- and Rooftop-Mounted Urban mmWave NR Deployments

**DOI:** 10.3390/s22030977

**Published:** 2022-01-27

**Authors:** Vyacheslav Begishev, Dmitri Moltchanov, Anna Gaidamaka, Konstantin Samouylov

**Affiliations:** 1 Department of Applied Probability and Informatics, Peoples’ Friendship University of Russia (RUDN University), 117198 Moscow, Russia; samuylov-ke@rudn.ru; 2 Unit of Electronics and Communications Engineering, Tampere University, 33100 Tampere, Finland; dmitri.moltchanov@tuni.fi (D.M.); anna.gaydamaka@tuni.fi (A.G.); 3 Laboratory of the Internet of Things and Cyber-Physical Systems, HSE University, 101000 Moscow, Russia; 4 Institute of Informatics Problems, Federal Research Center Computer Science and Control of Russian Academy of Sciences, 119333 Moscow, Russia

**Keywords:** millimeter wave, new radio, unmanned aerial vehicles, LoS blockage, closed-from approximation, rooftop deployments

## Abstract

Unmanned aerial vehicles (UAV) are envisioned to become one of the new types of fifth/sixth generation (5G/6G) network users. To support advanced services for UAVs such as video monitoring, one of the prospective options is to utilize recently standardized New Radio (NR) technology operating in the millimeter-wave (mmWave) frequency band. However, blockage of propagation paths between NR base stations (BS) and UAV by buildings may lead to frequent outage situations. In our study, we use the tools of integral geometry to characterize connectivity properties of UAVs in terrestrial urban deployments of mmWave NR systems using UAV line-of-sight (LoS) blockage probability as the main metric of interest. As opposed to other studies, the use of the proposed approach allows us to get closed-form approximation for LoS blockage probability as a function of city and network deployment parameters. As one of the options to improve connectivity we also consider rooftop-mounted mmWave BSs. Our results illustrate that the proposed model provides an upper bound on UAV LoS blockage probability, and this bound becomes more accurate as the density of mmWave BS in the area increases. The closed-form structure allows for identifying of the street width, building block and BS heights, and UAV altitude as the parameters providing the most impact on the considered metric. We show that rooftop-mounted mmWave BSs allow for the drastic improvement of LoS blockage probability, i.e., depending on the system parameters the use of one rooftop-mounted mmWave BS is equivalent to 6–12 ground-mounted mmWave BSs. Out of all considered deployment parameters the street width is the one most heavily affecting the UAV LoS blockage probability. Specifically, the deployment with street width of 20 m is characterized by 50% lower UAV LoS blockage probability as compared to the one with 10 m street width.

## 1. Introduction

The opportunities offered by unmanned aerial vehicles (UAVs) in a wide variety of fields have led to a dramatic increase in their production and deployment. Initially utilized in the military, UAVs today are applied in many fields including communications networks [1]. Specifically, UAVs can be used in wireless communications systems to reliably support connectivity in disaster management, public safety, and rescue operations [2,3,4].

The support of UAVs as a new network user in fifth generation (5G) systems opens up new opportunities related to the organization of services such as delivery, security surveillance, mapping navigation, and many others [5,6,7]. Furthermore, UAVs can be utilized by the network operator as repeaters and mobile base stations (BS). However, UAVs are characterized by the new unique properties compared to classic users (higher speed, higher position relative to the ground, etc.) and thus require new mechanisms to support them in 5G systems.

Many factors such as selected frequency range, line-of-sight (LoS) range, and signal attenuation play an important role in UAV communications. The work within 3GPP related to integration of UAV to 5G systems started with 3GPP SP-180909 (see Section 2 for a detailed overview of 3GPP standardization efforts) outlining requirements for communication delay, rate, and reliability as key performance indicators (KPI), for UAV applications. For example, security surveillance requires very high data rates in the downlink for air-to-ground communications [8]. Services such as private property monitoring, flying BSs, and mobile integrated access and backhaul (IAB) nodes also require high bandwidth at the air interface [9]. It is worth noting that some missions cannot be completed by one UAV. In such cases, a swarm of UAVs are needed, resulting in additional communications overheads [10]. Specifically, as a result of the movement of UAVs, the structure of the swarm may change dynamically, requiring regular updates.

Based on the abovementioned application requirements, UAVs need to be supported by all radio access technologies (RAT) within 5G systems. Within the range of technologies, the most challenging is the support of UAVs in the millimeter wave (mmWave) bands [11]. The rationale is that this band is highly susceptible to a blockage by buildings. A feasible solution to this problem would be to support the multi-connectivity functionality standardized for 5G NR systems [12]. According to it, when blockage occurs, it is possible to switch to another BS that is currently non-blocked. This technique has been shown to drastically improve performance of conventional terrestrial users, see, e.g., [13,14]. To assess the coverage of 5G mmWave NR deployments with multiconectivity functionality for UAV users, simple and accurate line-of-sight (LoS) blockage models are thus required [15].

The conventional approach to analyzing the coverage/outage phenomenon in the presence of blockage is to utilize the tools of stochastic geometry, see, e.g., [16,17,18] among others for human body blockage models. The core of the analysis is to estimate the probability that a LoS path between user equipment (UE) and BS is not blocked by obstacles having a certain shape. The major step is thus to determine the number of blockers falling to the so-called LoS blockage zone, see [19] for details. The approach has proved itself as a versatile tool for analysis of human blockage in mmWave systems with purely random deployments of blockers, where the dimensions of obstacles are negligible compared to the length of the path between communicating entities.

Analyzing regular deployments, where dimensions of the obstacles are not negligible as compared to the LoS path between the communicating entities, the described approach results in a number of inherent limitations. In particular, the probability that a LoS path is blocked by an obstacle depending on relative positions of obstacles with respect to each other leading to complex expressions for coverage/outage probabilities that cannot be provided in closed-form. When dealing with such deployments the results are often provided in product form either having infinite sums [20] or involving integration [21]. An alternative approach is to utilize field measurements of LoS blockage, see, e.g., [22]. The latter approach is mainly dictated by the simplicity of the final expression but is limited to those conditions where the measurements data have been gathered. Thus, there is a need for a model providing simple closed-form approximation for UAV LoS blockage probability accounting for both system and environment characteristics.

In this paper, we will target the abovementioned two challenges. Specifically, we first provide closed-form approximation for LoS blockage probability of UAVs in urban terrestrial deployments of mmWave systems. To this end, we utilize the tools of integral geometry rather than stochastic geometry. Then, we proceed to apply the proposed methodology to estimate the UAV LoS blockage probability in the rooftop deployment of BSs. The proposed approach allows for the providing of UAV LoS probability in closed-form in grounded, rooftop, and mixed grounded-rooftop deployments as a function of environmental characteristics.

Our main contributions can be summarized as follows:

- closed-form approximation for UAV LoS blockage probability in urban deployments of mmWave NR technology showing excellent agreement with complex models;
- numerical results showing that the most impact on UAV LoS blockage probability in ground-mounted mmWave deployment is produced by UAV altitude, BS height, street width, and mean building block height while the effect of other parameters is of secondary importance;
- numerical results for mixed ground-rooftop deployments of mmWave BSs showing that it allows for the drastic increase of UAV LoS blockage probability and, depending on system parameters, adding one rooftop-mounted mmWave BS is equivalent to adding 6–12 ground-mounted mmWave BSs.

The rest of the paper is organized as follows. First, we overview recent efforts in the analysis of UAV blockage probability in Section 2. The system model utilized in our study is introduced in Section 3. UAV LoS blockage probability for grounded and rooftop deployments is derived in Section 4. Numerical results are provided in Section 5. Conclusions are provided in the last section.

## 2. Related Work

In this section, we first review recent vendors’ and standardization bodies’ activities related to UAV integration into cellular 5G systems. We then proceed by providing an outlook of UAV LoS blockage models proposed over the last few years.

### 2.1. UAV Integration into 5G

In recent years, UAVs support in modern wireless networks has attracted attention from network operators and standardization organizations. The 3GPP TR 36.777 summarizes the research done on LTE support for UAVs. In particular, it considers several cellular network improvements for efficient service of UAV users, quantifies the impact of UAVs on the network, and evaluates characteristics of UAV-based service in urban and rural environments. Computer simulations of such systems, augmented with measurement data, show that the use of UAVs may lead to increased interference in both uplink and downlink directions. TR 36.777 also suggests methods to eliminate interference. Another issue identified in TR.36.777 is related to UAV mobility. The standard defines methods for providing additional information about the deployed ground network that can be used for decision-making during flight.

Since 3GPP Release 16, UAV support has been seen as a critical feature of the 5G cellular network infrastructure. In this context, TR 22.829 summarizes the use cases and analyzes UAV functions that may require enhanced support from access networks. It includes video broadcast applications, command and control services, and the use of UAVs as aerial BSs. The latter UAV application is covered in detail in TR 38.811.

3GPP is currently continuing research in this area. In particular, some of the tasks are to reduce the negative effects caused by the mobility of UAVs and to adapt to the needs of business, security, and the remote identification of UAVs. Specifically, TR 22.125 defines operational requirements for 3GPP systems. The 3GPP is expected to improve UAV integration methods in 5G communication networks in future revisions of TR 23.754 and TR 23.755. Nevertheless, it is already clear that UAVs will soon provide a wide range of services in 5G access networks.

### 2.2. LoS blockage Probability

The question of LoS occlusion by large static objects such as buildings has been significantly investigated in the context of terrestrial users. One of the fundamental studies dating back to 1984 [23] uses a methodology based on the combination of mathematical modeling and field measurements. Specifically, the study proposed a mathematical model describing a statistical method for predicting LoS propagation paths for a receiver-transmitter pair in densely populated areas based on a statistical building distribution model. The core of the model is based on an analysis of the mean free path of moving particles in randomly distributed obstacles. The resulting LoS blockage probability was calculated for a scenario where buildings are located along a certain axis between the receiver and transmitter, with building heights distributed exponentially.

The work in [24] includes a description of a model for calculating the LoS blockage probability for a pair UE-BS in the Fresnel zone of a certain radius, applicable to typical European cities with dense and regular streets. This empirical model is based on empirical data from the city center of Bristol, UK. The model takes into account the height of the buildings, their dimensions, the width of the streets, and the distribution of street corners. The carried out numerical analysis demonstrated that the distribution and variance of building height has little impact on the LoS blockage probability. Furthermore, in [16], a random shape theory for modeling random blockage effects in urban cellular networks is utilized. A fundamental method has been established to determine the LoS blockage probability from irregularly placed buildings. Although no direct comparison with empirical measurements has been performed, the main finding was that the LoS blockage probability decreases exponentially fast with the link length. Another example of a similar model for terrestrial users is reported in [25], where cube-shaped structures with uniformly distributed height are utilized as a model for buildings. The authors report the LoS blockage probability in integral form.

Recently, a number of models for UAV LoS blockage probability have been reported. In [26], the authors carried out a large-scale simulation campaign based on real data taken from the city of Ghent for collecting UAV coverage data with both LTE and mmWave BS terrestrial deployments. The reported data highlights that mmWave NR coverage of UAV is insufficient even for the highly dense deployment of these BSs. In [27], a method to estimate LoS blockage probability based on a scanning laser is proposed. This methodology is applied to open parking situations to collect data and use them to form an exponentially decaying probabilistic LoS blockage model.

Both ITU-R and 3GPP have also defined their LoS blockage models for UAV. In particular, the ITU-R model, reported in [20], considers the frequency range from 20 to 50 GHz. The LoS blockage probability is calculated assuming that the terrain is flat and has a certain constant slope over the area of interest. The model also accounts for different heights of UE and BS and uniform distribution of the building height. The LoS blockage probability is produced in product-form. Contrarily, 3GPP models of LoS blockage defined in TR 38.901 are purely empirical, obtained by fitting the measurement data to the exponentially decaying function starting from a certain breaking point. The model specifically tailored to UAV and proposed in TR.36.777 [28] utilizes only two parameters: BS height and UAV altitude. Parameters such as the height of buildings, building density and others are not taken into account. Thus, the model can only be used for certain BS heights, significantly reducing the application scenarios.

Recently, the authors in [21] proposed a detailed and versatile UAV LoS blockage probability that accounts for most critical parameters including different UAV and BS heights, different building height distribution, and various widths of streets and building blocks. The standard city deployment is however limited to the regular one and captured by the Manhattan Poisson line process (MPLP). Owing to the model complexity, closed-form expressions have been provided for specific building height distributions only. The authors demonstrated that the LoS blockage probability is highly sensitive to the type of deployment, the distribution of building heights, and the flight altitude of the UAV. Also, according to the authors, the existing standardized models developed by 3GPP and ITU-R provides an overly optimistic approximation of the UAV LoS blockage probability.

### 2.3. Summary

In summarizing, we note that the accuracy of empirical models proposed so far for UAV LoS blockage analysis heavily depends on the similarities of the analyzed deployment and the one where measurements have been taken. Specifically, measurement-based models require large-scale measurement campaigns for each specific environment. Purely analytical models are either too simple to account for critical details or do not provide the solution in closed-form.

In this paper, we will fill the abovementioned gap by proposing an accurate analytical model accounting for all the major specifics of the environment. The distinguishing feature of the proposed model is that, as opposed to other models, it provides the result in closed-form and is capable of capturing the specifics of both ground- and rooftop-mounted BS simultaneously.

## 3. System Model

In this section, we first introduce the considered system model by defining the system and environmental input parameters. We then define the metrics of interest and outline the proposed methodology.

### 3.1. Deployment Model and Metrics of Interest

**Figure 1:**
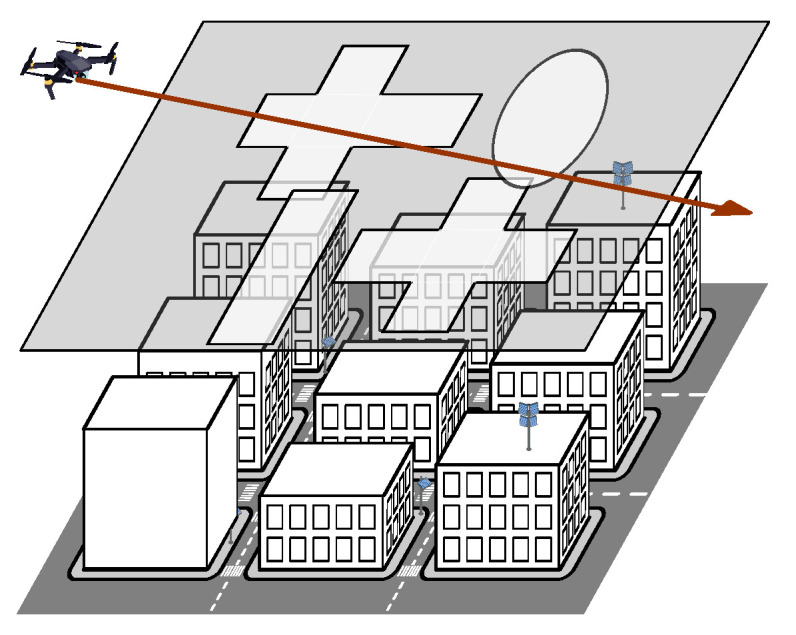
Illustration of the considered deployment.

We assume deterministic Manhattan grid deployment with street width *l*, see Figure 1. The widths and lengths of building blocks are assumed to be $b_{w}$ and $b_{l}$. The height of building blocks is assumed to be a random variable (RV), $H_{B}$, with probability density function (pdf) $f_{H_{B}}\left(x\right)$. We consider a certain zone of interest having $M_{V}$ and $M_{H}$ vertical and horizontal streets, respectively. We further assume that there are *N* ground-mounted mmWave BSs located on the streets leading to the spatial density of $N/[M_{V}*\left(l+b_{l}\right)*M_{H}\left(l+b_{w}\right)]$ mmWave BSs per squared meter. On top of this, we assume that there are *M* rooftop-mounted mmWave BS randomly located on the building roofs.

MmWave BSs are assigned to streets randomly, i.e., first a discrete uniformly distributed RV between 0 and $M_{H}+M_{V}$ is used to determine the street index, and then the position of mmWave BS is determined by choosing *x* or *y* coordinate uniformly along the street width, excluding parts occupied by crossroads. Similarly, $N_{C}$, $N_{C}<N$, crossroad-installed mmWave BSs are assigned to crossroads randomly using discrete RV uniformly distributed between 0 and $M_{V}M_{H}$. A particular location of mmWave BS on a crossroad is defined with respect to the left upper corner and is fully determined by the distances $l_{A,1}$ and $w_{A,1}$. Similarly, we choose a particular position for BSs installed along the street at the distance $l_{A,1}$, from the building. Note that in practice these BSs can be installed on lampposts, for example, and distances $l_{A,1}$, and $w_{A,1}$ may coincide with the sidewalk width.

The UAV attitude is assumed to be constant, $h_{R}$. We assume that UAV is in coverage of BS if there is a LoS path between UAV and BS and this path is less than a certain *r*. UAV is assumed to cross this region following a random line at the constant speed $v_{U}$. We are interested in the UAV coverage probability–the probability that UAV is in coverage of at least one BS.

### 3.2. Methodology at a Glance

Instead of accounting for inherent dependencies between building positions and their shapes in regular urban deployments, we characterize LoS visibility regions in ${ℜ}^{2}$ located at the UAV flying altitude, $h_{R}$, see Figure 1. Using these regions we then proceed by utilizing the tools of integral geometry to determine the probability that a random point in this plane is covered by at least one LoS visibility region immediately delivering the sought metrics of interest in a simple closed-form.

## 4. UAV Blockage Analysis

In this section, we develop our framework. We start by defining the so-called LoS visibility zones at the flying altitude of the UAV. Next, we utilize the integral geometry to specify the LoS probability for the ground deployment of mmWave BSs. Finally, we extend the methodology to account for rooftop-mounted mmWave BSs.

### 4.1. Geometric Structure of LoS Zones

**Figure 2:**
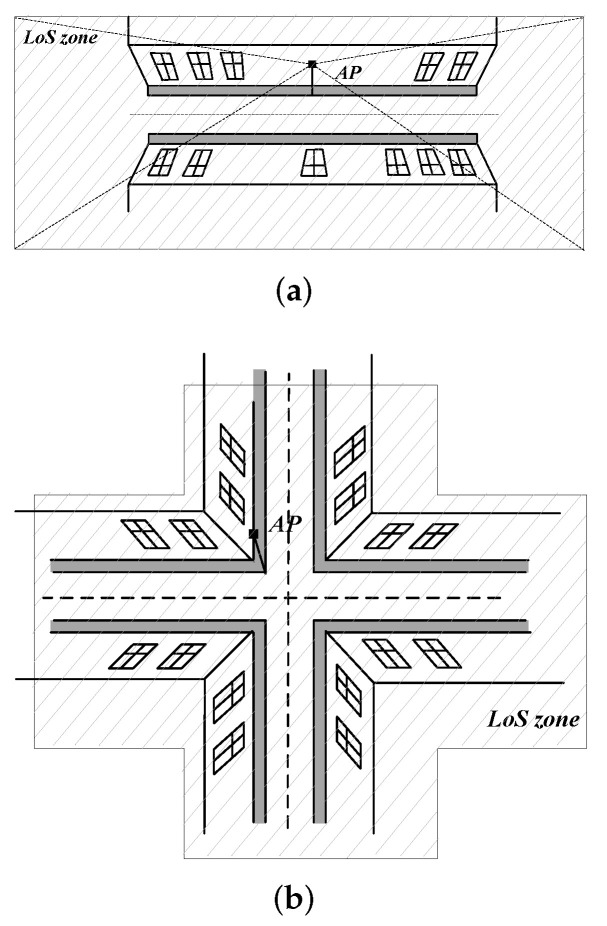
Two types of feasible LoS visibility zones in the considered scenario. (**a**) BS located along the street; (**b**) BS located at the crossroad.

We start by characterizing the LoS visibility zone induced by BR BS located along the street, see Figure 2a. As one may observe, this zone is of rectangular shape with sides that depend on (i) heights of buildings, $H_{B,1}$ and $H_{B,2}$, (ii) maximum coverage of BS, *r*, and (iii) UAV altitude $h_{R}$.

**Figure 3:**
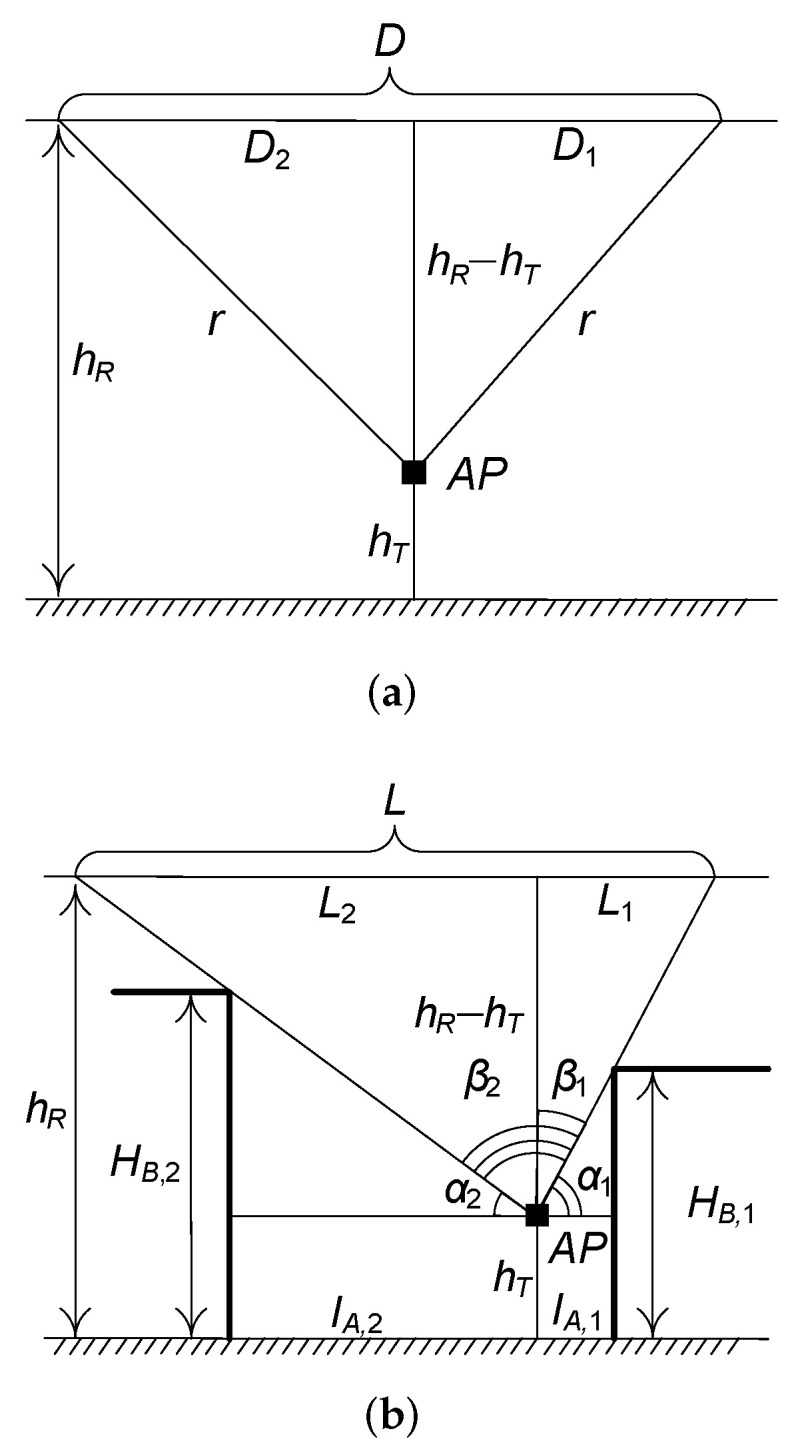
Geometrical illustration of the sides of LoS visibility zone. (**a**) Length of the LoS visibility zone; (**b**) Width of the LoS visibility zone.

Observing Figure 3a, the length of the LoS visibility zone is

(1) $$\begin{array}{c} D=2\sqrt{r^{2}-{\left(h_{R}-h_{T}\right)}^{2}}, \end{array}$$

where *r* is the maximum communications distance,

(2) $$\begin{array}{c} \;r=\sqrt{10^{\frac{P_{A}+G_{R}+G_{T}-N_{0}-S_{T}-32.4-20\log_{10}F_{C}}{21}}-{\left[h_{R}-h_{T}\right]}^{2}}, \end{array}$$

where $S_{T}$ is the SNR threshold, $G_{T}$ and $G_{R}$ are the transmit and receive antenna gains, $P_{A}$ is the emitted power at mmWave BS, $N_{0}$ is the thermal noise, $F_{C}$ is the carrier frequency.

The width of the LoS visibility zone, *L*, is an RV that is determined by building heights, $H_{B,1}$ and $H_{B,2}$, where both have the same pdf $f_{H_{B}}\left(x\right)$, see Figure 3b. Observe that angles $\alpha_{1}$ and $\alpha_{2}$ are given by

(3) $$\begin{array}{c} \alpha_{i}=\tan^{-1}\left(\frac{H_{B,i}-h_{T}}{l_{A,i}}\right),\;i=1,2, \end{array}$$

where $l_{1}$, $l_{2}$ are the distances to the buildings, see Figure 3b.

Further, using $\tan\beta_{i}=L_{i}/\left(h_{R}-h_{T}\right)$, i=1,2 and observing that angles $\beta_{i}$ are related to $\alpha_{i}$ as $\beta_{i}=\pi /2-\alpha_{i}$ we arrive at the following expressions for RVs $L_{1}$ and $L_{2}$

(4) $$\begin{array}{cc} L_{i} & =\left(h_{R}-h_{T}\right)\tan(\frac{\pi }{2}-\tan^{-1}[\frac{H_{B,i}-h_{T}}{l_{A,i}}])=\\ \; & =\frac{l_{A,i}\left(h_{R}-h_{T}\right)}{H_{B,i}-h_{T}},\;i=1,2. \end{array}$$

One may now determine the mean area of the LoS visibility zone as

(5) $$\begin{array}{cc} E[S_{B}] & =D\int_{0}^{\infty }\int_{0}^{\infty }f_{H_{B}}\left(x\right)f_{H_{B}}\left(y\right)\left[L_{1}\left(x\right)+L_{2}\left(y\right)\right]dxdy=\\ \; & =2\sqrt{r^{2}-{\left(h_{R}-h_{T}\right)}^{2}}E\left[L_{B}\right], \end{array}$$

where the mean length of the LoS visibility zone, $E[L_{B}]$, is provided by

(6) $$\begin{array}{cc} E[L_{B}] & =\int_{0}^{\infty }\int_{0}^{\infty }\left(\frac{l_{A,i}\left(h_{R}-h_{T}\right)}{x-h_{T}}\right)\times \\ \; & \times \left(\frac{l_{A,i}\left(h_{R}-h_{T}\right)}{y-h_{T}}\right)f_{H_{B}}\left(x\right)f_{H_{B}}\left(y\right)dxdy, \end{array}$$

that can be evaluated in closed-form for a given distribution of the building height.

A simple yet reliable approximation for (5) can be obtained by assuming the same random height of both buildings on the street, as it is usually the case in practice. In this case, the width of the blockage zone becomes

(7) $$\begin{array}{c} L_{B}=L_{1}+L_{2}=\frac{\left(h_{T}-h_{R}\right)\left(l_{A,1}+l_{A,2}\right)}{x-h_{T}}, \end{array}$$

implying that (6) can be written as

(8) $$\begin{array}{c} E\left[L_{B}\right]=\int_{0}^{\infty }f_{H_{B}}\left(x\right)\frac{\left(h_{T}-h_{R}\right)\left(l_{A,1}+l_{A,2}\right)}{x-hT}dx. \end{array}$$

For example, for $H_{B}$ having uniform distribution in (A,B) we have

(9) $$\begin{array}{c} E\left[L_{B}\right]=\frac{\left(h_{T}-h_{R}\right)\left(l_{A,1}+l_{A,2}\right)\left(\log\left[1-\frac{A}{h_{T}}\right]-\log\left[1-\frac{B}{h_{T}}\right]\right)}{B-A}. \end{array}$$

Similarly, the mean perimeter of the LoS visibility zone *B* is $E\left[L_{B}\right]=2D+2E\left[L\right]$. The LoS visibility zones induced by BS deployments on the crossroads can be found similarly. Indeed, as one may observe in Figure 2b, they consist of two overlapping LoS visibility zones forming a “cross”. Individually, parameters of these two zones can be estimated as shown above.

### 4.2. Blockage Probability with Grounded Infrastructure

We are now in a position to evaluate blockage probability, $p_{B}$, with ground-mounted BSs. The input parameters are the number of LoS visibility zones characterized by their mean areas and perimeters, $E[S_{B}]$ and $E[L_{B}]$, in ${ℜ}^{2}$ plane positioned at the UAV flying altitude $h_{R}$.

To provide simple yet accurate expression for blockage probability, we will rely upon the tools of integral geometry. Further, we need two fundamental notions of integral geometry. A curious reader is referred to [29] for a basic account of information and to [30] for modern developments in the field.

**Definition** **1** (**Kinematic density, [29].**) *Let K denote the group of motions of a set A in the plane. The kinematic density dA for the group of motions K in the plane for the set A is*

(10) $$\begin{array}{c} dA=dx\wedge dy\wedge d\phi , \end{array}$$

*where ∧ is the exterior product [31], x and y are Cartesian coordinates, ϕ is the rotation angle of A with respect to OX.*

**Definition** **2** (**Kinematic measure, [29].**) *The kinematic measure m of a set of group motions K on the plane is defined as the integral of the kinematic density dA over K, that is,*

(11) $$\begin{array}{c} m_{A}=\int_{K}dA=\int_{K}dx\wedge dy\wedge d\phi . \end{array}$$

Consider first a single mmWave BS in the area of interest *A* and let *B* define a LoS visibility zone. We are first interested in the probability $p_{C}$ that UAV, located at a randomly chosen point *P* in *A*, is in coverage of this BS, that is, it is located in *B*. Using conditional probability we may write

(12) $$\begin{array}{c} p_{C}=\frac{Pr\{P\in A\cap B\}}{Pr\{A\cap B\neq 0\}}, \end{array}$$

where the probability that UAV location *P* belongs to the intersection area of two sets, *A* and *B*, is in the nominator, while the probability that these sets do intersect is in the denominator.

Using the notion of kinematic measure, we get [29]

(13) $$\begin{array}{cc} \; & Pr\{P\in A\cap B\}=m(A:P\in A\cap B\}),\\ \; & Pr\{A\cap B\neq 0\}=m(A:A\cap A\neq 0), \end{array}$$

where the first expression is the kinematic measure of the set of motions of *A* such that P∈A, while the second one provides the measure of all motions of *A*, for which the intersection between *A* and *B* is non-zero.

Following [29], the first measure is

(14) $$\begin{array}{cc} \; & m_{j}\left(P\in A\cap B\right\})=\int_{P\in B}f\left(x,y\right)dx\wedge dy\wedge d\phi , \end{array}$$

where f(x,y) is the density of LoS visibility zone positions in *A*.

The measure of all motions of *A*, such that A∩B, is [29]

(15) $$\begin{array}{cc} m_{j}\left(A\cap B\neq 0\right) & =\int_{A\cap B\neq 0}f\left(x,y\right)dx\wedge dy\wedge d\phi . \end{array}$$

Finally, the sought probability is given by

(16) $$\begin{array}{c} p_{C}=\frac{\int_{P\in A\cap B}f\left(x,y\right)dx\wedge dy\wedge d\phi }{\int_{A\cap B\neq 0}f\left(x,y\right)dx\wedge dy\wedge d\phi }, \end{array}$$

and can be computed for a particular form of *A*, *B*, and f(x,y).

The numerator in (16) is computed as [29]

(17) $$\begin{array}{cc} m_{j}\left(P\in A\cap B\right\}) & =\int_{P\in B}dx\wedge dy\wedge d\phi =\\ \; & =\int_{P\in B}\;dx\wedge dy\int_{0}^{2\pi }\;d\phi =2\pi E\left[S_{B}\right], \end{array}$$

where $E[S_{B}]$ is the mean area of LoS visibility zone provided in (5).

The measure of motions of *A* is such that A∩B≠0 is [29]

(18) $$\begin{array}{cc} m_{j}\left(A\cap B\neq 0\right) & =\int_{A\cap B\neq 0}dx\wedge dy\wedge d\phi =\\ \; & =2\pi \left(S_{A}+E\left[S_{B}\right]\right)+L_{A}E\left[L_{B}\right], \end{array}$$

where $E[L_{B}]$ is the perimeter of LoS visibility zone, $S_{A}$ and $L_{A}$ are the area and the perimeter of *A*, given by

(19) $$\begin{array}{cc} \; & S_{A}=\left[M_{V}*\left(l+b_{l}\right)+b_{l}\right]*\left[M_{H}\left(l+b_{w}\right)+b_{l}\right],\\ \; & L_{A}=2[M_{V}*\left(l+b_{l}\right)+b_{l}]+2\left[M_{H}\left(l+b_{w}\right)+b_{l}\right]. \end{array}$$

Substituting (17), (18) into (12) we obtain

(20) $$\begin{array}{c} p_{C}=\frac{2\pi E[S_{B}]}{2\pi \left(S_{A}+E\left[S_{B}\right]\right)+L_{A}E\left[L_{B}\right]}. \end{array}$$

Recall that mmWave BSs are deployed randomly along the streets. When mmWave BS is deployed on the crossroad it creates two LoS visibility zones as illustrated in Figure 3b. Let *u* be the probability that mmWave BS is at the crossroad. This probability is found as the ratio of crossroad area to the overall area of streets as

(21) $$\begin{array}{c} u=\frac{M_{V}M_{H}l^{2}}{M_{H}\left(l\left[\left(b_{w}+l\right)M_{V}+b_{w}\right]\right)+M_{V}\left(l\left[\left(b_{l}+l\right)M_{H}+b_{l}\right]\right)-M_{V}M_{H}l^{2}}. \end{array}$$

The mean number of LoS visibility zones of rectangular shape is then given by the mean of Binomial distribution with parameters *N* and *u* shifted by *N*, i.e., N(1+u). Thus, the blockage probability can now be approximated as

(22) $$\begin{array}{c} p_{B}=1-{\left(1-p_{C}\right)}^{N(1+u)}, \end{array}$$

where $p_{C}$ is provided in (20).

Substituting intermediate results and simplifying, we arrive at the closed-form expression for blockage probability in the presence of *N* ground-mounted mmWave BS as

(23) $$\begin{array}{c} p_{B}=1-{\left(1-\frac{2\pi E[S_{B}]}{2\pi \left(S_{A}+E\left[S_{B}\right]\right)+L_{A}E\left[L_{B}\right]}\right)}^{N(1+\frac{M_{V}M_{H}l^{2}}{M_{H}\left(l\left[\left(b_{w}+l\right)M_{V}+b_{w}\right]\right)+M_{V}\left(l\left[\left(b_{l}+l\right)M_{H}+b_{l}\right]\right)-M_{V}M_{H}l^{2}})}. \end{array}$$

where $S_{A}$ and $L_{A}$ are provided in (19), $E[S_{B}]$ and $E[L_{B}]$ are calculated using (5) and (6) for a given $f_{H_{B}}\left(x\right)$.

### 4.3. Blockage Probability with Rooftop-Mounted BSs

The blockage probability heavily depends on the density of mmWave BSs, as well as on the heights of buildings. For some values of these input parameters, the blockage probability might be unacceptably high. In practical deployments, network operators may want to add additional dedicated mmWave BSs. Mounting these BSs on rooftops would allow for an unobstructed LoS of circular shape, drastically reducing blockage probability.

To assess joint deployment, one may apply the methodology developed in the previous section to rooftop mmWave BSs. The principal difference is that the LoS visibility zone is of circular form with radius D/2 as in (1) with $H_{B}$ replacing $h_{T}$. However, as these mmWave BSs are now deployed on the roofs, *D* is a RV. Thus, we have

(24) $$\begin{array}{c} E\left[D\right]=\int_{0}^{\infty }f_{H_{B}}\left(x\right)2\sqrt{r^{2}-{\left(h_{R}-x\right)}^{2}}dx. \end{array}$$

that can be evaluated for a given $f_{H_{B}}\left(x\right)$, $x<h_{R}$.

The blockage probability by *M* rooftop mmWave BSs is obtained similarly to (22). Finally, in the presence of *N* ground-mounted and *M* rooftop-mounted mmWave BSs the blockage probability is the product of individual blockage probabilities.

## 5. Numerical Results

In this section, we first assess the accuracy of the model identifying its application range, and then proceed to report on the impact of system parameters on the UAV blockage probability. Finally, we evaluate the effect of rooftop-mounted BSs. The values of input system parameters are provided in Table 1.

### 5.1. Accuracy Assessment

**Figure 4:**
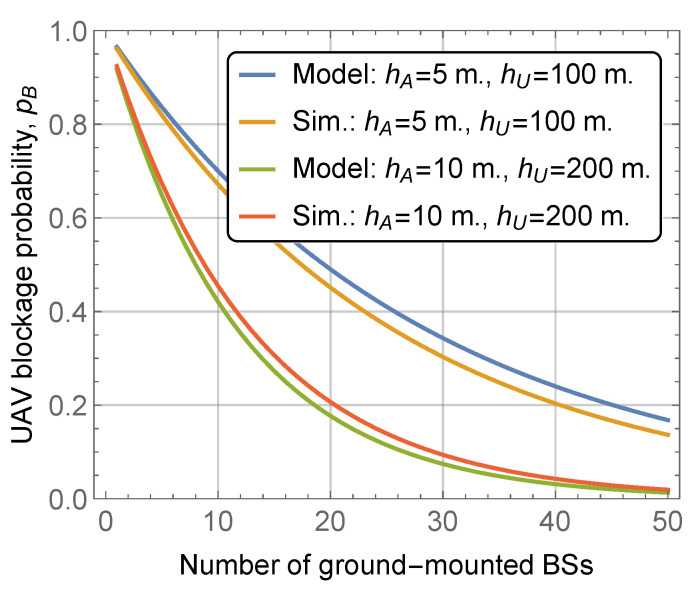
Comparison of the developed model and computer simulations.

To identify the application range of the developed closed-form approximation, we start assessing the accuracy of the model by comparing its results to those obtained using the computer simulations. To this end, Figure 4 shows the UAV LoS blockage probability obtained using the proposed model and computer simulations for various UAV altitudes and BS heights $h_{U}$ and $h_{A}$, respectively, street width l=20 m, mean building height and standard deviation $E[H_{B}]=30$ m and $\sigma [H_{B}]=10$ m, block width and length of $b_{w}=b_{l}=100$ m. The considered region of interest is formed by considering 10 horizontal and vertical building blocks interchanged with streets.

By analyzing the results shown in Figure 4, one may deduce that the proposed model allows for the approximation of the results obtained via computer simulations quite closely. Similar observations have been made for rooftop-mounted mmWave BSs. Notably, the developed model slightly overestimates the actual value of the probability. This is explained by the inherent structure of the model that assumes that all the LoS visibility regions are completely independent. This observation allows us to identify the applicability regions of the model. First of all, observe that due to the abovementioned property the model always provides the upper bound on the UAV LoS blockage probability. Secondly, the results become more accurate as of the area of the zone and/or the density of the mmWave BSs increase. Based on these results, when discussing the response of the UAV blockage probability to system parameters and assessing the effect of rooftop-mounted mmWave BSs, we thus utilize the developed model.

### 5.2. Effects of System Parameters

We now proceed to evaluating the effect of system parameters on the UAV blockage probability including the BS height and altitude of UAV, the mean and variance of building height and, finally, the street width and building block’s width and length.

**Figure 5:**
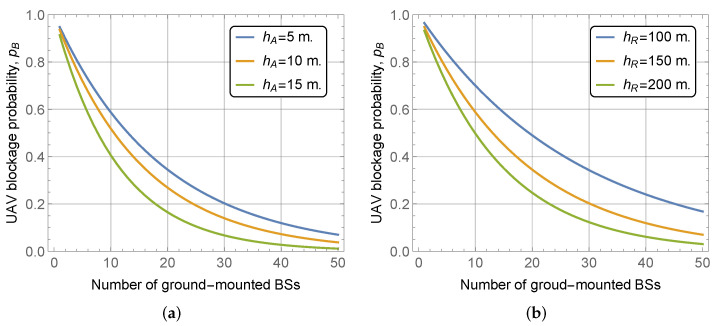
UAV blockage probability as a function of BS height and UAV altitude. (**a**) Various BS heights; (**b**) Various UAV altitudes.

We start with an assessment of the effects of mmWave height and UAV flying altitude. To this aim, Figure 5 shows UAV LoS blockage probability as a function of these parameters for street width l=20 m, mean building height and standard deviation $E[H_{B}]=30$ m and $\sigma [H_{B}]=10$ m, block width and length of $b_{w}=b_{l}=100$ m. By analyzing the presented results, we see that higher BS heights result in lower UAV LoS blockage probability, see Figure 5a. Particularly, the gain of changing mmWave BS height from 5 m to just 15 m leads to the decrease of UAV LoS blockage probability by approximately 0.15 for 20 mmWave BS deployed in the area. The rationale for these improvements is that higher mmWave BS heights make the visible regions at the UAV flying altitude larger, see Figure 2. Furthermore, this effect is non-linear as the area increases faster when the mmWave BS height increases. We also note that these gains depend heavily on BS deployment density and are minimal highly dense deployments.

Analyzing the effect of UAV flying altitude in Figure 5b, qualitatively similar conclusions can be made. More specifically, the higher the altitude the smaller the UAV LoS blockage probability. Specifically, for the density of 20 mmWave BS in the considered area, the gain of changing the altitude from 100 to 200 m is approximately 0.15 and is comparable to that of the change in BS height from 5 to 15 m. We also note that in practice this parameter should be tuned with care. The reason is that higher altitudes may lead to much lower received power, especially for ground-mounted mmWave BS that is usually downtilted to provide better coverage for terrestrial users, e.g., pedestrians.

**Figure 6:**
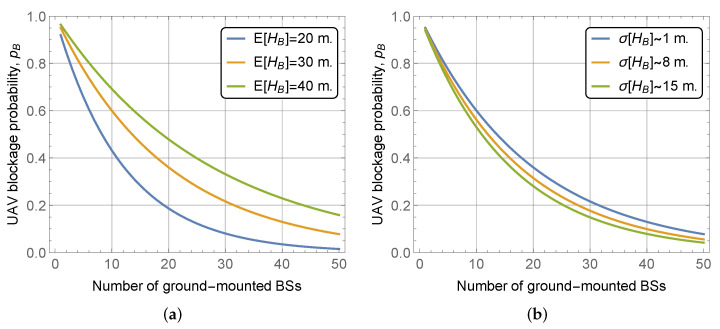
UAV blockage probability as a function of building block height parameters. (**a**) Various mean heights; (**b**) Various standard deviations.

In dense city deployments of mmWave BS, the characteristics of building block height may produce a significant impact on UAV LoS blockage probability. We investigate this hypothesis in Figure 6, where we illustrate the UAV LoS blockage probability as a function of the number of deployed mmWave BS for UAV altitude $h_{U}=150$ m, BS height $h_{A}=5$ m, street width l=20 m, block width and length of $b_{w}=b_{l}=100$ m. Here, in Figure 6a we show the effect of different mean values by keeping the standard deviation constant at $\sigma [H_{B}]=10$, while in Figure 6b we vary standard deviation and keep the mean constant at $E[H_{B}]=30$ m.

By analyzing the presented data, we may conclude that the mean building height logically produces a significant effect on the UAV LoS blockage probability. The magnitude of this effect is comparable to that of BS height or UAV altitude. Particularly, when considering districts with high building heights, e.g., city centers, one needs to utilize additional ways to improve UAV LoS blockage probability. However, at the same time, the effect of standard deviation is rather limited, leading to differences in the range of 0.05–0.1 for the considered range of the number of deployed mmWave BS.

**Figure 7:**
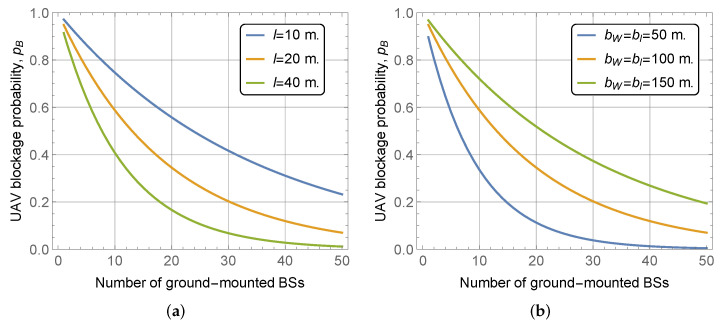
UAV blockage probability as a function of street and building block widths. (**a**) Various street widths; (**b**) Various building block width.

Finally, we consider the effect of street and building block widths on UAV LoS blockage probability illustrated in Figure 7 for UAV altitude $h_{U}=150$ m, BS height $h_{A}=5$ m, mean building height and standard deviation $E[H_{B}]=30$ m and $\sigma [H_{B}]=10$ m, respectively, block width and length of $b_{w}=b_{l}=100$ m. As one may observe, both parameters drastically affect the considered metric of interest. However, the effects are different. Specifically, by increasing the street width the UAV LoS blockage probability drastically increases, see Figure 7a. The rationale is that this leads to much larger areas of LoS visibility zones, see Figure 2. At the same time, one may observe that by increasing the street and building block widths, the considered area increases as the number of streets and building blocks in both horizontal and vertical directions are kept constant. Thus, logically, larger building blocks dimensions lead to higher UAV LoS blockage probability, see Figure 7b. Nevertheless, this effect is attributed to the increase of the considered area.

### 5.3. The Effect of Rooftop-Mounted BSs

**Figure 8:**
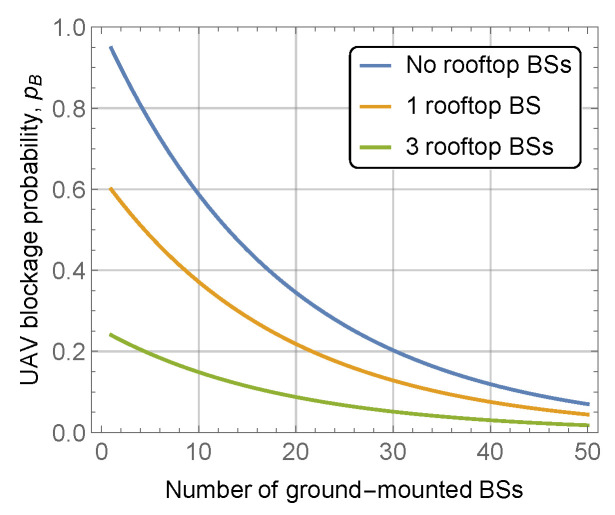
The effect of the rooftop-mounted BSs.

Finally, we highlight the effect of rooftop-mounted BS on the UAV blockage probability. To this aim, Figure 8 shows the effect of rooftop-mounted mmWave BSs on the UAV blockage probability for UAV altitude $h_{U}=150$ m, BS height $h_{A}=5$ m, street width l=20 m, mean building height and standard deviation $E[H_{B}]=30$ m and $\sigma [H_{B}]=10$ m, block width and length of $b_{w}=b_{l}=100$ m. By analyzing the presented data, one may observe that mounting BSs on rooftops allows us to greatly reduce the BS blockage probability. More specifically, adding just three rooftop-mounted mmWave BSs to the considered area allows for the reduction of the UAV LoS blockage probability by multiple times. Recall that in the considered deployment the deployment area is $\left(b_{l}+l\right)M_{V}\times \left(b_{w}+l\right)*M_{H}\approx 1.44\times 10^{6}$ m${}^{2}$, implying that the density of rooftop BS is just ≈ 2 $\times 10^{-6}$ BS/km${}^{2}$. Specifically, by comparing the horizontal and vertical distances between lines in Figure 8, we observe that in terms of UAV LoS blockage probability, adding additional BS at the rooftop is equivalent to deploying 10 more ground-mounted mmWave BSs. This value is affected by system parameters and environmental characteristics of the deployment and may vary between six and twelve.

### 5.4. Discussion, Limitations and Applications

The presented results illustrate that out of all considered deployment parameters, street width and building block length are the ones impacting the UAV LoS blockage probability the most. The impact of BS and UAV heights as well as the mean building block height is also noticeable. These parameters all need to be accounted for when estimating the required density of BSs to support UAVs in mmWave 5G systems. Note that in real deployments, these parameters are not independent as specified in [20]. Thus, in general, in city centers, where the mean building heights and width are larger, much higher BS deployment density will be required for the same target UAV LoS blockage probability as compared to the suburbs.

We specifically emphasize the importance of rooftop-mounted BS. As we have observed, qualitatively, the density of ground-mounted BS deployment has to be extremely high, especially in city center deployment conditions. Here, to support the uninterrupted connectivity, it is much more economically sustainable for network operators to deploy dedicated BSs having almost unobstructed coverage for UAV. Our results demonstrate that one rooftop-mounted BS is equivalent to six to twelve ground-mounted ones in terms of UAV LoS blockage probability.

Although the proposed model by design can capture the specifics of different deployments, it also has its limitations. Specifically, as the model assumes that visibility areas are all convex, the visibility areas created by BSs deployed on the crossroads need to be treated as two independent rectangular visibility areas. This implies that the accuracy of the model increases as the size of the analyzed regions with homogeneous building deployments increases. Furthermore, the independence of all visibility areas also implies that the BS locations should be close to the Poisson point process (PPP, [32]). Note that due to restrictions of BS locations in the city center and also due to the need for high densification to satisfy the growing customer needs, BS deployment locations are far from regular cellular structures. Specifically, many studies assume PPP as the deployment process for 4G/5G systems.

The proposed model is especially usable in system-level simulations of mmWave NR deployments supporting UAVs. As noticed in [33], the handling of dynamic blockage events is one of the most time-consuming operations. Associating UAVs with the blockage process having the fraction of time in blockage coinciding with the UAV LoS blockage probability may efficiently address this challenge. Furthermore, the proposed model can be utilized by network operators at the network deployment phase to assess the density of mmWave BSs providing the required level of UAV coverage.

## 6. Conclusions

UAVs are expected to soon become a vital part of 5G deployments, acting as both users and aerial BSs. Motivated by the use of UAVs in future 5G deployments, in this paper, we utilize the tools of integral geometry to provide closed-form approximations for UAV blockage probability. In addition to LoS blockage with ground-mounted mmWave BSs, we also considered the case of the operator utilizing rooftop-mounted mmWave BSs.

The numerical results illustrate that the model can closely match the actual UAV LoS blockage probability. Furthermore, the accuracy of approximation increases as either the density of mmWave BSs or the area of interest increases. In analyzing the effect of the rooftop-mounted mmWave BSs, we have shown that one additional rooftop-mounted BS improves the UAV LoS blockage probability as six to twelve ground-mounted mmWave BS. Finally, the most impact on UAV blockage probability is produced by the mmWave BS height, UAV altitude, street width, and mean building block height. The developed model allows for the mathematical assessment of the sought metric for a given deployment condition and density of ground- and rooftop-mounted mmWave BSs.

We foresee two application areas of the proposed model. The first is with regard to system-level simulations, where one needs to utilize simple models for UAV LoS blockage probability. Additionally, the model can be utilized for assessment of the required density of mmWave NR BS to ensure a certain UAV LoS blockage probability. We also note that the accuracy of the model increases as the deployment area with the homogeneous building deployments increases. Thus, the proposed model needs to be applied to large city districts.

## Figures and Tables

**Table 1:** Summary of notation and parameters.

Parameter	Value
mmWave BS height, $h_{T}$	5 m
UAV height, $h_{R}$	150 m
Carrier frequency, $F_{C}$	28 GHz
Emitted power, $P_{T}$	0.02 W
mmWave BS and UAV antenna gains, $G_{T},G_{R}$	15 dB, 5 dB
Number of vertical and horizontal streets, $M_{H},M_{V}$	10, 10
Length and width of building blocks, $b_{l},b_{w}$	100 m, 100 m
Street width, *l*	20 m
SNR threshold, $S_{T}$	0 dB
Thermal noise, $N_{0}$	−174 dBm

## Data Availability

Not applicable.
